# Cardiac complication after experimental human malaria infection: a case report

**DOI:** 10.1186/1475-2875-8-277

**Published:** 2009-12-03

**Authors:** An-Emmie Nieman, Quirijn de Mast, Meta Roestenberg, Jorien Wiersma, Gheorghe Pop, Anton Stalenhoef, Pierre Druilhe, Robert Sauerwein, André van der Ven

**Affiliations:** 1 Department of Medical Microbiology, Radboud University Nijmegen Medical Center, Nijmegen, The Netherlands; 2 Department of General Internal Medicine, Radboud University Nijmegen Medical Center, Nijmegen, The Netherlands; 3 Department of Cardiology, Radboud University Nijmegen Medical Center, Nijmegen, The Netherlands; 4 Unité de Parasitologie Biomédicale, Institut Pasteur, Paris, France

## Abstract

A 20 year-old healthy female volunteer participated in a clinical Phase I and IIa safety and efficacy trial with candidate malaria vaccine *Pf*LSA-3-rec adjuvanted with aluminium hydroxide. Eleven weeks after the third and last immunization she was experimentally infected by bites of *Plasmodium falciparum-*infected mosquitoes. When the thick blood smear became positive, at day 11, she was treated with artemether/lumefantrine according to protocol. On day 16 post-infection i.e. two days after completion of treatment, she woke up with retrosternal chest pain. She was diagnosed as acute coronary syndrome and treated accordingly. She recovered quickly and her follow-up was uneventful. Whether the event was related to the study procedures such as the preceding vaccinations, malaria infection or antimalarial drugs remains elusive. However, the relation in time with the experimental malaria infection and apparent absence of an underlying condition makes the infection the most probable trigger. This is in striking contrast, however, with the millions of malaria cases each year and the fact that such complication has never been reported in the literature. The rare occurrence of cardiac events with any of the preceding study procedures may even support a coincidental finding.

Apart from acute coronary syndrome, myocarditis can be considered as a final diagnosis, but the true nature and patho-physiological explanation of the event remain unclear.

## Background

Experimental human malaria infections are a well accepted method for testing efficacy of candidate pre-erythrocytic malaria vaccines since 1971 [1-5]. The clinical presentation of experimental human malaria infections is generally mild and associated with symptoms of uncomplicated malaria [1,2]. The most commonly reported symptoms and laboratory abnormalities are headache, malaise, fever, thrombocytopaenia and leucopaenia. With close monitoring and immediate administration of treatment upon detection of parasitaemia, experimental human malaria infections have been considered safe and well-tolerated [1-3].

Here, a cardiac event occurring shortly after treatment is described in a healthy volunteer participating in a malaria vaccine phase I/IIa trial.

## Case presentation

A 20-year old, healthy female volunteer (medical student) participated in a double blind randomized phase I conditional IIa trial with the candidate malaria vaccine *Pf*LSA-3-rec in 2007-2008. The volunteer's medical history was unremarkable, except for a tonsillectomy at the age of 4 and mild atopic symptoms for which she occasionally used a nasal corticosteroid. As medication, she also used a second generation oral contraceptive. Family history revealed a myocardial infarction in the paternal grandfather, when 43 years old, and a positive paternal family history for dyslipidemia. She never smoked or used illicit drugs. At inclusion, the blood pressure was 135/88 mmHg with a pulse of 64/minute and a body mass index of 20 kg/m^2^; the results of the physical examination were normal. Subsequent blood pressure measurements yielded values below 130/80 mmHg. ECG and laboratory tests were normal, including plasma glucose and lipid levels (Table 1).

**Table 1:** Clinical Laboratory findings

Haematology and biochemistry tests	Reference range	Screening	Admission	Adm +1	Adm +2	Adm +3	Adm +4 = Discharge
Haemoglobin (mmol/l)	7.3 - 9.7	7.1	6.9		7.1		6.9

Leukocytes (*10^9^/l)	3.5 - 11.0	6.6	7.9		3.9		5.4

Platelets (*10^9^/l)	120 - 350	259	160		254		302

Glucose (mmol/l)	4.0 - 5.5 (fasting)	4.6	4.9				

Sodium (mmol/l)	137 - 144	142	140		137		

Potassium (mmol/l)	3.4 - 4.6	4.1	3.9		3.9		

Creatinin (umol/l)	45 - 90	61	55		60		

Urea nitrogen (mmol/l)	3.0 - 7.0		4.9		3.2		

Bilirubin (umol/l)	≤ 17	<10				<10	

Alkaline phosphatase (U/l)	≤ 120	55	74		66	69	69

Aspartate aminotransferase (U/l)	≤ 40	16	132	104	111	89	83

Alanine aminotransferase (U/l)	≤ 45	15	137	134	171	166	164

Lactate dehydrogenase (U/l)	≤ 450	313	540	534	562		384

gamma GT (U/l)	≤ 35	13	29		39	36	35

Creatinin Kinase (U/l)	≤ 170		296	105			28

Troponin I (ug/l)	≤ 0.20		10.40	2.75			0.20

CRP (mg/l)	≤ 10		26				

Cholesterol (mmol/l)	4.7 - 6.5		4.0				

Triglycerides (mmol/l)	0.8 - 2.0		1.5				

HDL cholesterol (mmol/l)	1.10 - 1.70		1.20				

LDL cholesterol (mmol/l)	≤ 3.5		2.12				

							

**Coagulation assays**							

INR	0.9-1.1		1.0	1.0			

Fibrinogen (mg/l)	2000-4000			4320			

Factor VIII activity (%)	60-150			130%			

Protein C activity (%)	70-150			>100%			

Free protein S antigen (%)	55-115			92%			

Homocystein (μmol/l)	< 13.4			9.3			

Antithrombin III (%)	85-120			98%			

Factor II mutation	negative			negative			

Factor V (Leiden) mutation	negative			negative			

von Willebrand Factor (%)	50-150		166%				

ADAMTS13 activity (%)	60-140		90%				

							

**Additional tests**							

Convalescent microbiological tests	adenovirus, RS-virus, influenza virus, mycoplasma pneumonia, Chlamydia, Q-fever, para-influenza, enterovirus						Negative
Auto-immune antibodies	ANCA, anticardiolipin antibodies, β2 glycoprotein, lupus anticoagulans, anti-heart (rat) antibodies						Negative
Toxicology test	tetrahydrocannabinol, amphetamine-derivatives, cocaine, diazepam, methadone, tramadol, opiates						Negative
							
Inflammatory parameters	Interleukin 6, Interleukin 8						< detection limit

							

Time after last dose	7 h15						103 h45
Artemether	< detection limit						< detection limit
Dihydroartemisinin	26.5 ng/ml						< detection limit
Lumefantrine	4308 ng/ml and						443 ng/ml
Desbutyl-lumefantrine	40 ng/ml						18 ng/ml

The volunteer received three immunizations with 30 μg of LSA-3 recombinant protein adjuvanted with aluminium hydroxide. Immunizations were administered subcutaneously in the upper arm at monthly intervals. Adverse events after immunization were injection site pain, and headache with increasing intensity after each immunization. The headache after the third vaccination was severe and accompanied by photophobia; it lasted for two days and made her keep bed rest.

Permission from the Independent Research Committee was obtained for proceeding with the phase IIa part of the trial. Eleven weeks after the last immunization the volunteer was exposed to the bites of five mosquitoes infected with *Plasmodium falciparum *(strain NF54). The thick blood smear became positive eleven days after exposure (44 parasites/μl, infected red blood cells 0.0012%) and treatment with 80/480 mg artemether/lumefantrine (Riamet^®^) was immediately initiated. The dose was repeated after 8, 24, 36, 48 and 60 hours. At the time of treatment onset the volunteer reported headache, fatigue and myalgia. These symptoms had started at day eight and nine after exposure. The day after treatment onset the volunteer reported intermittent fever, which had completely resolved two days later. The highest recorded sublingual temperature was 39.6°C. Artemether/lumefantrine was continued until day 14 and blood smears and PCR were negative on this day. On day 15 she experienced some shortness of breath when performing physical exercise, which she attributed to hay fever.

On day 16, two days after completion of treatment and with a negative blood smear, the volunteer woke up at 5.30 a.m. with retrosternal chest pain characteristic of angina pectoris, not radiating, not related to respiration or change of position. Pain was severe during 30 minutes and at that time accompanied by nausea. The volunteer called the study doctor who referred her to the cardiologist. On admission, the volunteer appeared not in acute distress, blood pressure was 120/80 mmHg, pulse 75/min, oxygen saturation 100% while breathing ambient air and temperature was 37.9°C. The jugular venous pressure was normal, as was the examination of the heart, lungs and peripheral arteries. The electrocardiogram showed 1 mm ST elevation in leads II, III and AVF and a negative T-wave in lead V2 (see Additional file 1, 2, 3, 4). A limited rise and fall of troponin I (peak 11,8 μg/l on day of admission) and other cardiac enzymes, including CPK, LDH and AST was found. The results of laboratory investigations are presented in Table 1.

On admission, chest X-ray was normal and the echocardiogram showed mild hypokinesia of the inferior wall. Cardiac MRI performed four days after start of the complaints did not show any abnormalities. A second cardiac MRI after ten months conducted in a specialized center in the Netherlands showed absence of aberrant coronaries and good cardiac function with small patchy sub-epicardial staining (posterior)-laterally suggestive for myocarditis (see Additional file 5). A coronary angiography was not performed.

The volunteer was admitted to the cardiac care unit with a diagnosis of acute coronary syndrome. Treatment consisted of nitroglycerin, metoprolol, perindopril, calcium carbasalate, clopidrogel, enoxaparine and acetaminophen. Complaints completely resolved within 30 minutes after treatment and the further clinical course was uneventful, e.g. the patient was free of symptoms throughout a follow-up period of one year. After four days, the volunteer was discharged from the hospital in good condition. Medications were gradually discontinued after discharge; only calcium carbasalate was continued for six months. Given the MRI data and evolution so far the prognosis is currently considered good.

## Discussion

A case of limited myocardial necrosis occurring just after completion of anti-malarial treatment is reported in an experimentally infected healthy volunteer without obvious risk factors for cardiovascular disease. A relationship between the cardiac event and the parasite challenge and/or its treatment seems probable, especially because of the chronology of the event and the absence of an alternative explanation. Remarkably however, cardiac complications are extremely rare in malaria. To date, myocardial infarction during or after naturally acquired *P. falciparum *infection has not been reported, neither in hundreds of millions of endemic cases, nor in hundreds of thousands military troops that were temporarily deployed to endemic areas [6-8]. In Sierra Leone, a patient was described with angina pectoris and a positive blood slide, which may have been an incidental finding in an highly endemic area [9]. Elevated troponins without cardiac complaints or ECG abnormalities are rare; an elevation just above threshold was measured in 1 out of 161 malaria patients [10,11]. Finally, a few cases of myocarditis have been reported and only during severe or fatal *falciparum *malaria [12-15].

Regarding a possible effect of the anti-malarial treatment, it should be noted that the cardiac event occurred shortly after therapeutic administration of artemether/lumefantrine. There is no direct evidence for ischemic cardiotoxicity of this drug combination [16] and artemether, dihydroartemisinin and lumefantrine concentrations in the volunteer, measured in the Centre Hospitalier Universitaire Vaudois in Lausanne, Switzerland, were within therapeutic ranges (Table 1). Desbutyl-lumefantrine levels were elevated according to Hatz and Lefebvre, however according to McGready these values are normal [17-19]. One case of coronary artery spasm was reported in a 17-year old male using artemether/lumefantrine, but that patient's urine was also tested positive for tetrahydrocannabinol, the active substance in marijuana, which may possibly contribute to this complication [20].

Since 1985, experimental human malaria infections have been conducted in three centers in 1367 volunteers. No cardiac complications have been reported, except a case of myocardial infarction in 2002 whereby a 39-year old male volunteer developed a myocardial infarction during malaria treatment by chloroquine, two days following detection of malaria parasites in a blood smear [2,21], i.e. with a similar chronology as in the above reported case. However, this volunteer had cardiovascular risk factors (hypercholesterolaemia and smoking) and the angiography, which was performed in this case, revealed a stenosis of one of the coronary arteries. In retrospect, the reality of a true parasitaemia was debatable since blood samples tested by RT-PCR several months later proved to be negative [2].

A plausible diagnosis in the LSA-3 volunteer is an acute coronary syndrome, since limited myocardial necrosis was found as expressed by the small rise and fall of cardiac enzymes including the most specific one, troponin. Also chest pain occurred that was mimicking angina pectoris and that disappeared after treatment with nitrates. Finally temporary changes on ECG and echocardiography were found, both corresponding for damage in the inferior wall. An acute coronary syndrome can occur in patients with normal or with abnormal coronary arteries.

An acute coronary syndrome associated with coronary atherosclerosis primarily occurs in patients over the age of 40. Young patients with myocardial infarction secondary to atherosclerosis usually have multiple risk factors, such as dyslipidemia, hypertension, smoking, diabetes mellitus, obesity and a positive family history [22,23]. In this volunteer, the only risk factors present is a positive family history, although not in a first degree family member. Anomalous coronary arteries are a rare cause of myocardial infarction in young patients [24]. There were, however, no indications for coronary abnormalities by cardiac MRI.

Myocardial ischaemia may also occur in patients with normal coronary arteries. Patients with this condition are generally younger, female and have no history of angina pectoris or traditional risk factors for atherosclerosis, except smoking [25,26]. Although the exact pathogenic mechanism often remains elusive, various mechanisms may contribute, e.g. coronary vasospasm, arterial thrombosis and hypercoagulability, and inflammation [27].

Variant angina, also Prinzmetal's angina, is caused by coronary vasospasm and might be a possible explanation for the symptoms in this volunteer. The classic patient is a young woman without traditional cardiovascular risk factors, except for tobacco use [28]. In some patients, variant angina is a manifestation of a more general vasospastic disorder and associated with Raynaud's phenomenon and migraine [29]. Although the volunteer has no previous history of a vasospastic disorder, she experienced severe migraine-like headache with photophobia after the last immunization, which may theoretically have been caused by increased vasospastic sensitivity. Vasospasm may also be induced by illicit drugs, most notably cocaine, but also marijuana [30,31]. The volunteer denied to have used recreational drugs and the toxicology screening was negative.

Coronary thrombosis, often secondary to an inherited or acquired thrombophilia, may also cause a transient coronary obstruction. No inherited thrombophilia was detected in this volunteer. Acquired thrombophilia may result from changes in coagulation cascade, endothelial function and platelets. Hypercoagulability caused by auto-immune disease, e.g. the antiphopholipid syndrome, seems unlikely because antiphospholipid antibodies and the ANCA were negative (Table 1).

Inflammation may result in a transient coagulopathy, although in this volunteer neither a pronounced pro-inflammatory state, nor a pronounced coagulopathy were observed [32]. The volunteer did use an oral contraceptive which might increase the risk for arterial thrombosis, although this risk is minimal in young, non-smoking women [23].

Inflammation also affects endothelial cell function. Endothelial dysfunction may impair endothelium-dependent relaxation, induce vasoconstriction and vasospasm and promote platelet adhesion [33,34]. Release of von Willebrand factor (vWF) may mediate this platelet adhesion [35]. Endothelial cell activation with adherence of infected erythrocytes is considered a key factor in the pathogenesis of a *P. falciparum *infection. Indeed, the occurrence of endothelial cell activation with release of active vWF has previously been demonstrated in early experimental malaria infections [36]. Increasing evidence suggests a potential mechanistic role for vWF in the pathogenesis of acute coronary syndromes[37]. However, significant rises in vWF levels have also been demonstrated in patients with naturally acquired malaria without the development of any coronary syndromes [38].

Nonetheless, regardless of biological explanations, various studies have found recent infections to be associated with acute coronary syndromes with normal coronary arteries[39].

A substantial fall in platelet numbers is observed during the *P. falciparum *blood stage in experimental malaria, followed by a rapid recovery and a transient relative thrombocytosis [2,36]. Young platelets are considered to be more reactive and increased numbers of young platelets have been reported in patients with myocardial infarction [40,41].

Finally, histamine is a strong inducer of endothelial cell activation. The volunteer has an allergic diathesis and the day before the event she experienced some shortness of breath, which she attributed to hay fever. In the Kounis syndrome acute coronary syndromes are associated with mast cell activation and hypersensitivity [42,43].

Changes in endothelial cell function, platelets and the coagulation system during experimental malaria might increase the likelihood for an acute coronary syndrome. However, it should be noted again that these changes also occur in naturally acquired malaria infections in individuals living in malaria-endemic regions and in non-immune travelers - both groups in whom acute coronary syndromes are not encountered.

Another possible diagnosis in this young female is myocarditis. Differentiation between acute coronary syndrome and myocarditis is difficult since many clinical and laboratory findings overlap. Because of the young age, the absence of clear cardiovascular risk factors and the findings by cardiac MRI, myocarditis is a reasonable alternative diagnosis. A high occurrence of myocarditis has been found in young patients without risk factors presenting with a clinical picture of an acute coronary syndrome and normal coronary arteries [44]. Post-infectious myocarditis can be associated with cardiac events especially in combination with viral infections [45-49]. Convalescent titers in the patient were negative, neither confirming nor excluding viral infection.

In relation to malaria few cases of myocarditis have been reported, but these reports were limited to patients with severe or fatal *falciparum *malaria [12-15]. In uncomplicated malaria, with low parasitaemia, as was the case in this volunteer, myocarditis has never been reported. Vaccinations, in particular smallpox have been associated with myocarditis, though in a small number of cases [49-52]. The clinical presentation of post-vaccination myocarditis may mimic that of an acute coronary syndrome, making the diagnosis particularly challenging [48,53]. In this volunteer, the event occurred rather late at 94 days after the last LSA3 immunization, which makes the association less likely. In such case, a boost of anti-LSA3 antibodies by the malaria challenge would have been expected, which was not detected.

## Conclusion

In conclusion, this volunteer presented with limited myocardial necrosis after an experimental human malaria infection. Multiple factors possibly contributing to the cardiac event can be identified, e.g. the preceding vaccinations, malaria infection or antimalarial drugs. However, the relation of this serious adverse event with the experimental malaria infection is probable because of its relation in time and apparent absence of underlying illness; this is in striking contrast, however, with the millions of malaria cases each year and the fact that such complication has never been reported in literature. The rare occurrence of cardiac events with any of the preceding study procedures may even support a coincidental finding. Apart from acute coronary syndrome, myocarditis can be considered as final diagnosis but the true nature and patho-physiological explanation of the event remain unclear.

## Consent

Informed consent was obtained from the patient for publication of this case report. A copy of the written consent is available for review by the Editor-in-Chief of this journal.

## Competing interests

The authors declare that they have no competing interests.

## Authors' contributions

AEN, QDM, MR and AVDV were clinical investigators, JW involved in execution of the trial, GP consultant cardiology, AS consultant vascular medicine, PD project coordinator, RS principle investigator, AEN, QDM, MR, AVDV and RS wrote the paper with comments from the other authors, All authors read and approved the manuscript.

## Supplementary Material

Additional file 1 Electrocardiogram showing the electrocardiograms previous to admission (number 1, 05 Sept 2007), whilst having oppressive, non-radiating pain on the chest (number 2, 28 Feb 2008 8:26 hrs) and after treatment (number 3, 28 Feb 2008 21:06 hrs and number 4, 03 March 2008). Click here for file

Additional file 2 Electrocardiogram showing the electrocardiograms previous to admission (number 1, 05 Sept 2007), whilst having oppressive, non-radiating pain on the chest (number 2, 28 Feb 2008 8:26 hrs) and after treatment (number 3, 28 Feb 2008 21:06 hrs and number 4, 03 March 2008). Click here for file

Additional file 3 Electrocardiogram showing the electrocardiograms previous to admission (number 1, 05 Sept 2007), whilst having oppressive, non-radiating pain on the chest (number 2, 28 Feb 2008 8:26 hrs) and after treatment (number 3, 28 Feb 2008 21:06 hrs and number 4, 03 March 2008). Click here for file

Additional file 4 Electrocardiogram showing the electrocardiograms previous to admission (number 1, 05 Sept 2007), whilst having oppressive, non-radiating pain on the chest (number 2, 28 Feb 2008 8:26 hrs) and after treatment (number 3, 28 Feb 2008 21:06 hrs and number 4, 03 March 2008). Click here for file

Additional file 5 **Cardiac Magnetic Resonance Images**. MRI images (file MRI.gif), showing the MRI image made ten months after the event. Late contrast enhanced pictures show a small, patchy staining sub-epicardial laterally and postero-laterally and slight staining spots on the infero-septal - inferior border. Click here for file
