# Supplementary figures and images for: Cardiac complication after experimental human malaria infection: a case report
Source: Malar J. 2009 Dec 3;8:277. doi: 10.1186/1475-2875-8-277 (PMC2794284; doi:10.1186/1475-2875-8-277)

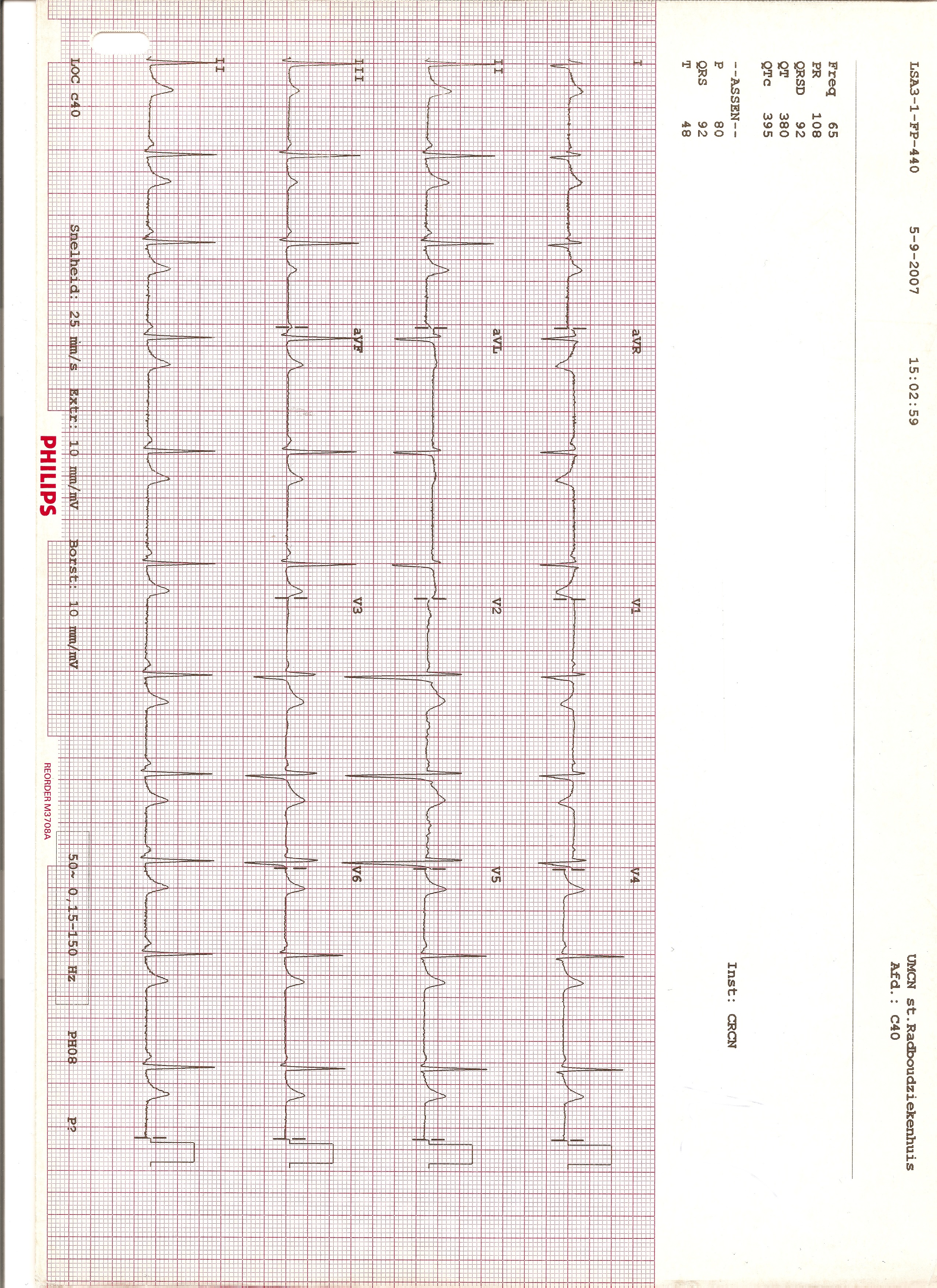

Supplement: Additional file 1 — Electrocardiogram showing the electrocardiograms previous to admission (number 1, 05 Sept 2007), whilst having oppressive, non-radiating pain on the chest (number 2, 28 Feb 2008 8:26 hrs) and after treatment (number 3, 28 Feb 2008 21:06 hrs and number 4, 03 March 2008). [file 1475-2875-8-277-S1.JPEG]

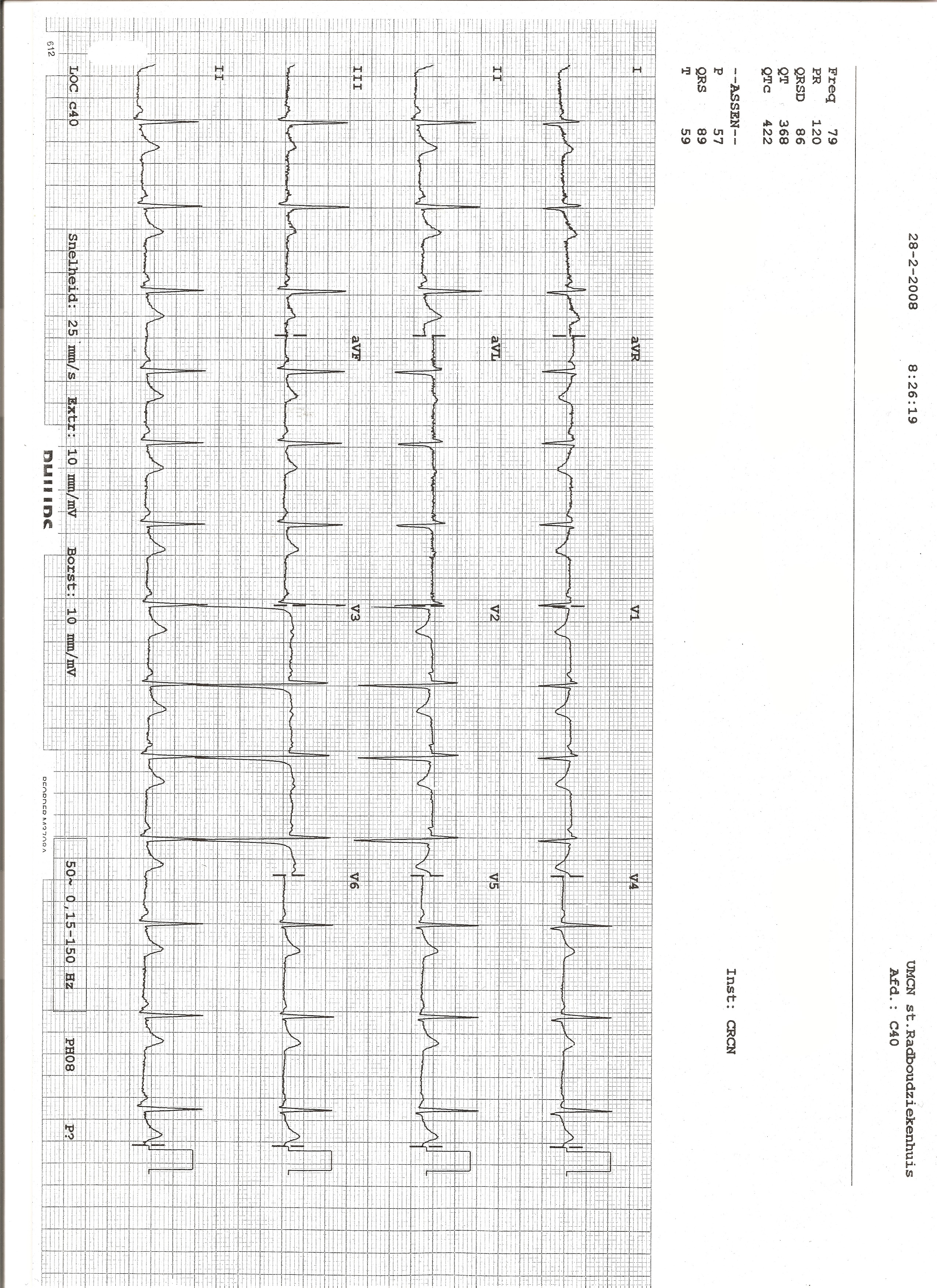

Supplement: Additional file 2 — Electrocardiogram showing the electrocardiograms previous to admission (number 1, 05 Sept 2007), whilst having oppressive, non-radiating pain on the chest (number 2, 28 Feb 2008 8:26 hrs) and after treatment (number 3, 28 Feb 2008 21:06 hrs and number 4, 03 March 2008). [file 1475-2875-8-277-S2.JPEG]

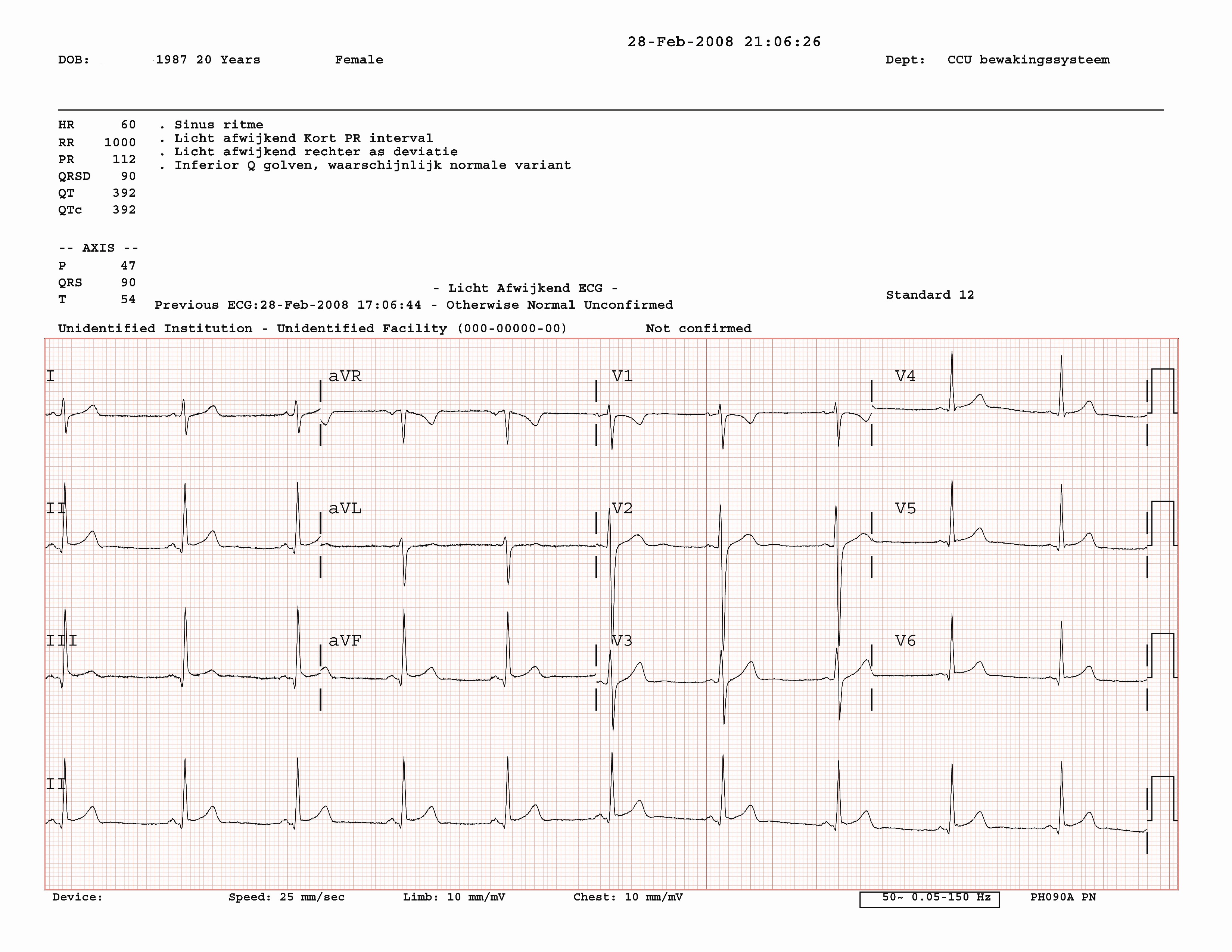

Supplement: Additional file 3 — Electrocardiogram showing the electrocardiograms previous to admission (number 1, 05 Sept 2007), whilst having oppressive, non-radiating pain on the chest (number 2, 28 Feb 2008 8:26 hrs) and after treatment (number 3, 28 Feb 2008 21:06 hrs and number 4, 03 March 2008). [file 1475-2875-8-277-S3.JPEG]

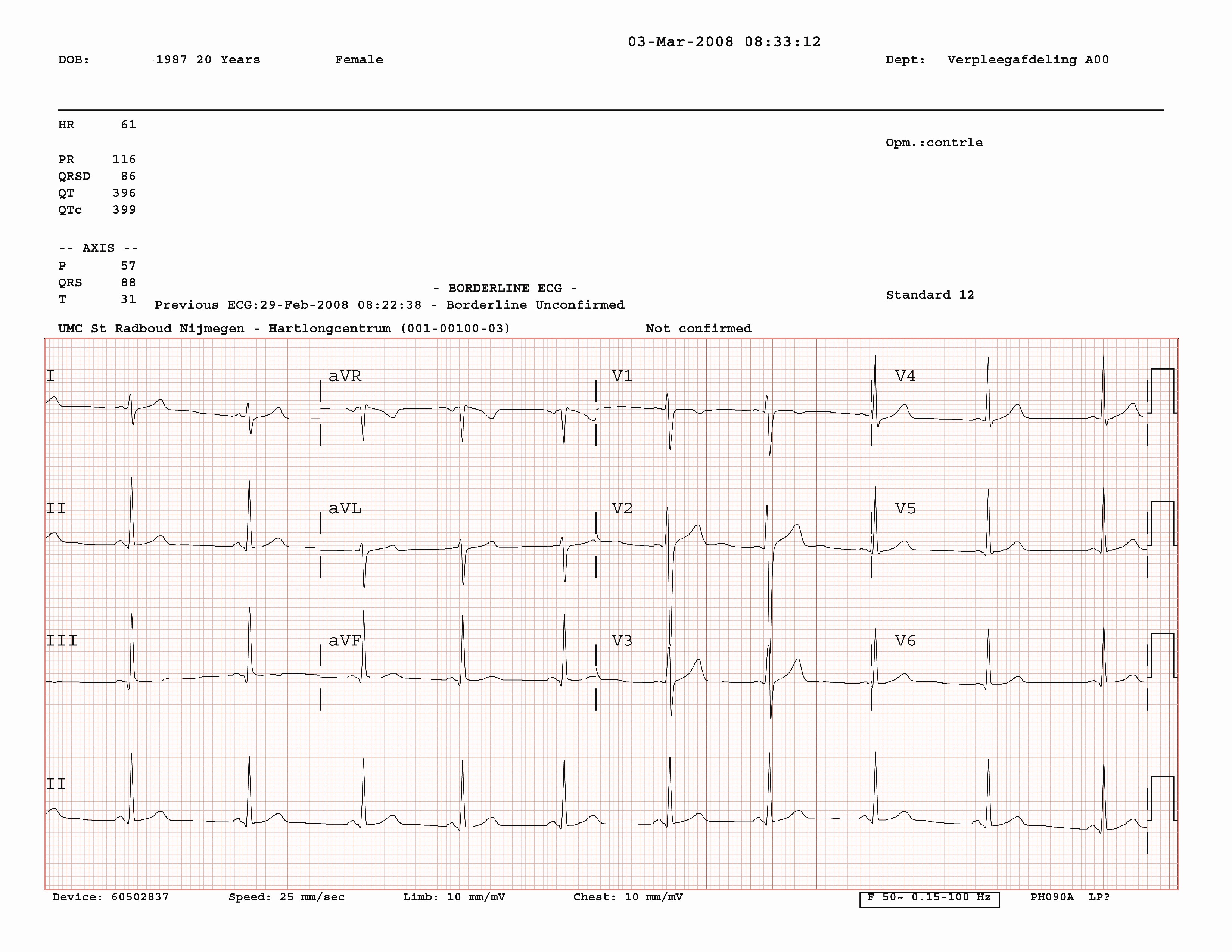

Supplement: Additional file 4 — Electrocardiogram showing the electrocardiograms previous to admission (number 1, 05 Sept 2007), whilst having oppressive, non-radiating pain on the chest (number 2, 28 Feb 2008 8:26 hrs) and after treatment (number 3, 28 Feb 2008 21:06 hrs and number 4, 03 March 2008). [file 1475-2875-8-277-S4.JPEG]
